# Development and evaluation of an immunochromatographic assay to detect serum anti-leptospiral lipopolysaccharide IgM in acute leptospirosis

**DOI:** 10.1038/s41598-017-02654-8

**Published:** 2017-05-23

**Authors:** Galayanee Doungchawee, Direk Sutdan, Kannika Niwatayakul, Tasanee Inwisai, Athisri Sitthipunya, Naphatsawan Boonsathorn, Titipatima Sakulterdkiat, Worachart Sirawaraporn, Visith Thongboonkerd

**Affiliations:** 1 Department of Pathobiology, Faculty of Science, Mahidol University, Bangkok, Thailand; 2 Thawangpha Hospital, Nan, Thailand; 3 Loei Hospital, Loei, Thailand; 4 Regional Medical Science Center 7, Khon Kaen, Thailand; 5 Center of Excellence for Vectors and Vector-Borne Diseases, Faculty of Science, Mahidol University, Salaya Campus, Nakhon Pathom Thailand; 6 Medical Proteomics Unit, Office for Research and Development, Faculty of Medicine Siriraj Hospital, Mahidol University, Bangkok, Thailand; 7 Center for Research in Complex Systems Science, Mahidol University, Bangkok, Thailand

## Abstract

Leptospirosis is a common life-threatening disease worldwide. However, its diagnosis is frequently ineffective because the gold standard bacterial culture and microscopic agglutination test (MAT) are usually positive 1–2 weeks after the disease onset. We thus developed an immunochromatographic assay (LEPkit) to detect serum anti-leptospiral lipopolysaccharide (LPS) IgM for rapid diagnosis of acute leptospirosis. Using referenced sera of 77 leptospirosis and 91 non-leptospirosis cases, LEPkit yielded 97.4% sensitivity, 94.5% specificity, 93.8 positive predictive value (PPV), 97.7% negative predictive value (NPV), and 95.8% accuracy. The stability of this kit stored for up to 18 months and its reproducibility were confirmed. Testing in 74 new cases using samples at admission-phase and subsequent paired samples (total n = 135), overall sensitivity was 98.5%, whereas that of culture and single MAT (≥1:400) was 15.6% and 35.6%, respectively. When only the samples at admission-phase were used (n = 74), the sensitivity remained at 98.7%, whereas that of culture and single MAT (≥1:400) was 28.4% and 13.5%, respectively. In summary, our LEPkit was far more effective than any conventional methods for the diagnosis of acute leptospirosis, especially within the first few days after the disease onset. The ease of use, stability and reproducibility further enhance its feasibility for clinical use on-site.

## Introduction

Leptospirosis is a life-threatening zoonosis caused by *Leptospira* of which more than 250 serovars have been recognized^1^. The global importance of this infectious disease has been evidenced by an approximation of 500,000 cases reported annually around the globe with a rising trend^2–9^. In addition, the majority of these cases have severe clinical manifestations and poor outcome due to a lack of rapid test, which is sensitive and effective enough for early detection that prompts proper intervention^10–12^. Moreover, clinical manifestations of leptospirosis are too generalized (e.g. fever, headache and myalgia) that are non-specific and difficult to differentiate from other prevalent and endemic infectious diseases with similar symptoms, i.e. dengue virus infection, malaria, melioidosis and scrub typhus^13–18^, especially those co-infected with dengue virus in endemic areas^19–21^. Because of the under-recognition of leptospirosis and the lack of suitable rapid diagnostic tool, its precise incidence/prevalence and burden are still unclear.

There have been a number of several attempts to employ enzyme-linked immunosorbent assay (ELISA)^22–30^ and lateral flow assay^31, 32^ for the diagnosis of acute leptospirosis. However, their sensitivity was not satisfactory (17.5–86%), especially for the detection of acute-phase samples collected within 7 days after the disease onset. Although several other approaches have implemented recombinant leptospiral proteins (e.g., LipL32, LipL41, OmpL1, and rLig) as the test antigens, their sensitivity was quite low with varying specificity^27, 29, 30, 33–36^. Hence, the early diagnosis of acute-phase leptospirosis remained handicapped due to a lack of effective rapid test for such early detection.

Recent evidence has shown that leptospiral lipopolysaccharide (LPS), especially at low molecular mass range (i.e. 10–30 kDa), could be detected by leptospirosis patients’ sera using immunoblotting^37–39^. We thus developed an in-house immunochromatographic rapid test kit imprinted with leptospiral LPS to detect anti-leptospiral LPS IgM in sera of patients with acute leptospirosis. Its sensitivity, specificity, positive predictive value (PPV), negative predictive value (NPV), and accuracy were evaluated in 77 known cases of acute leptospirosis diagnosed by gold standard test and 91 negative controls. Its reproducibility and stability after storage at room temperature for up to 18 months were evaluated. Finally, its application for detection of acute leptospirosis in 74 new cases was evaluated comparing to the gold standard test.

## Results

### Initial assessment of anti-leptospiral LPS IgM antibody in patients’ sera for detection of leptospiral LPS by IgM immunoblotting

**Figure 1:**
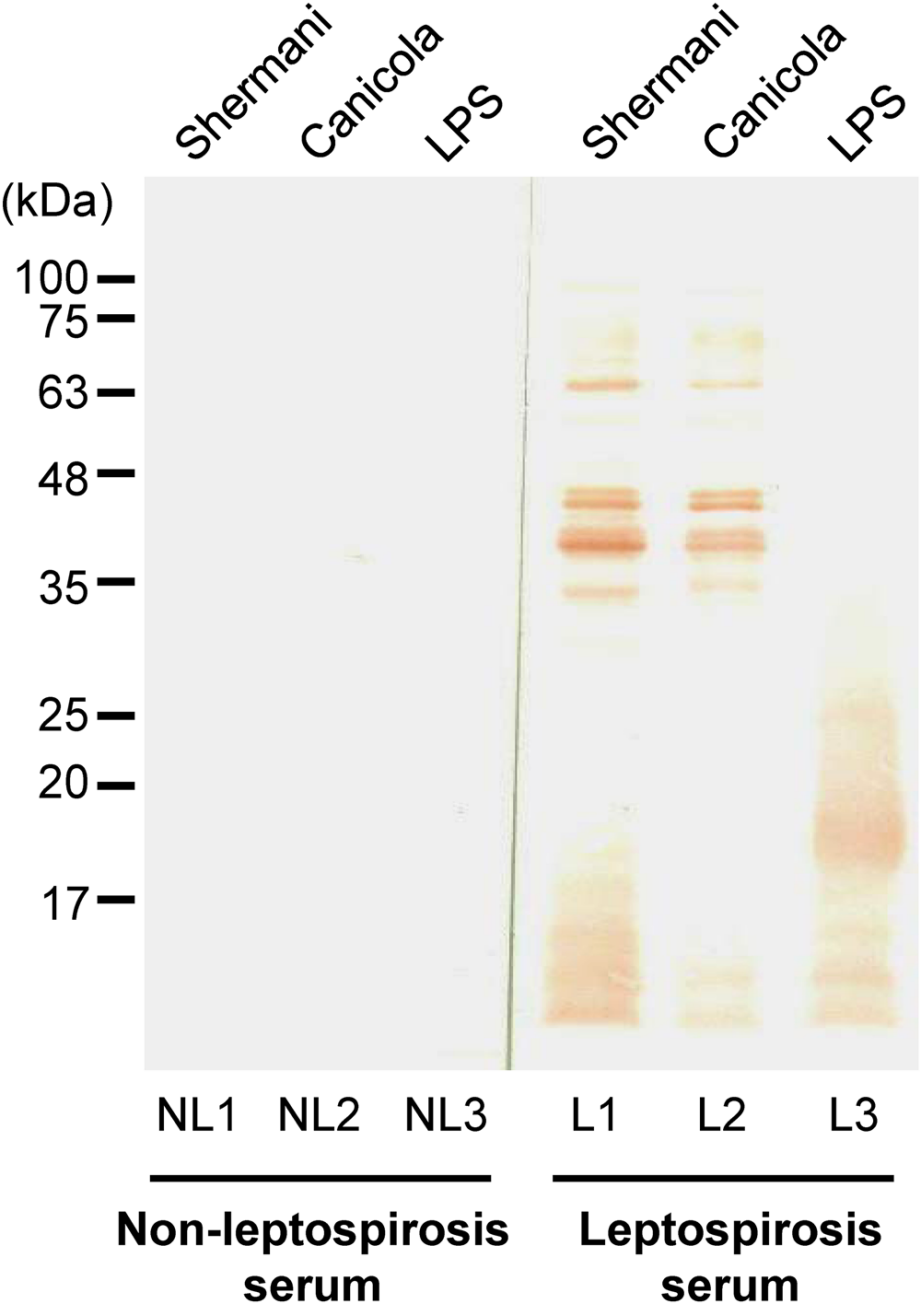
IgM immunoblotting. Leptospiral whole cell lysate derived from serovars Shermani and Canicola as well as the extracted LPS were resolved by 12% SDS-PAGE in lanes #1, #2 and #3, respectively. After blotting, the membrane was incubated with serum from non-leptospirosis (NL) or leptospirosis (L) patient, and then with rabbit anti-human IgM conjugated with horseradish peroxidase. The immunoreactive bands were visualized using 3,3′-diaminobenzidine (DAB).

The specificity and antigenic potential of the LPS extract were evaluated prior to implementation to the diagnostic kit assembly. Whole cell lysates derived from *Leptospira* serovars Shermani and Canicola, as well as LPS derived from the six combined *Leptospira* serovars of local prevalence (including Autumnalis, Bratislava, Canicola, Pomona, Sejroe and Shermani) were resolved by 12% SDS-PAGE and transferred onto a PVDF membrane. After incubation with serum from acute leptospirosis or non-leptospirosis patient followed by rabbit anti-human IgM conjugated with horseradish peroxidase, the immunoreactive bands were visualized by 3,3′-diaminobenzidine (DAB). The data revealed strong and consistent immunoreactivity of IgM in serum from leptospirosis patient to detect antigens from whole cell lysate of the leptospires, whereas the intense bands were detected at low molecular mass range (<30 kDa) using LPS as the antigens (Fig. 1). Additionally, there was no cross reactivity of the serum from non-leptospirosis patient (Fig. 1).

### Quality assessment of the LEPkit on detection of acute leptospirosis with low and high MAT titers

**Figure 2:**
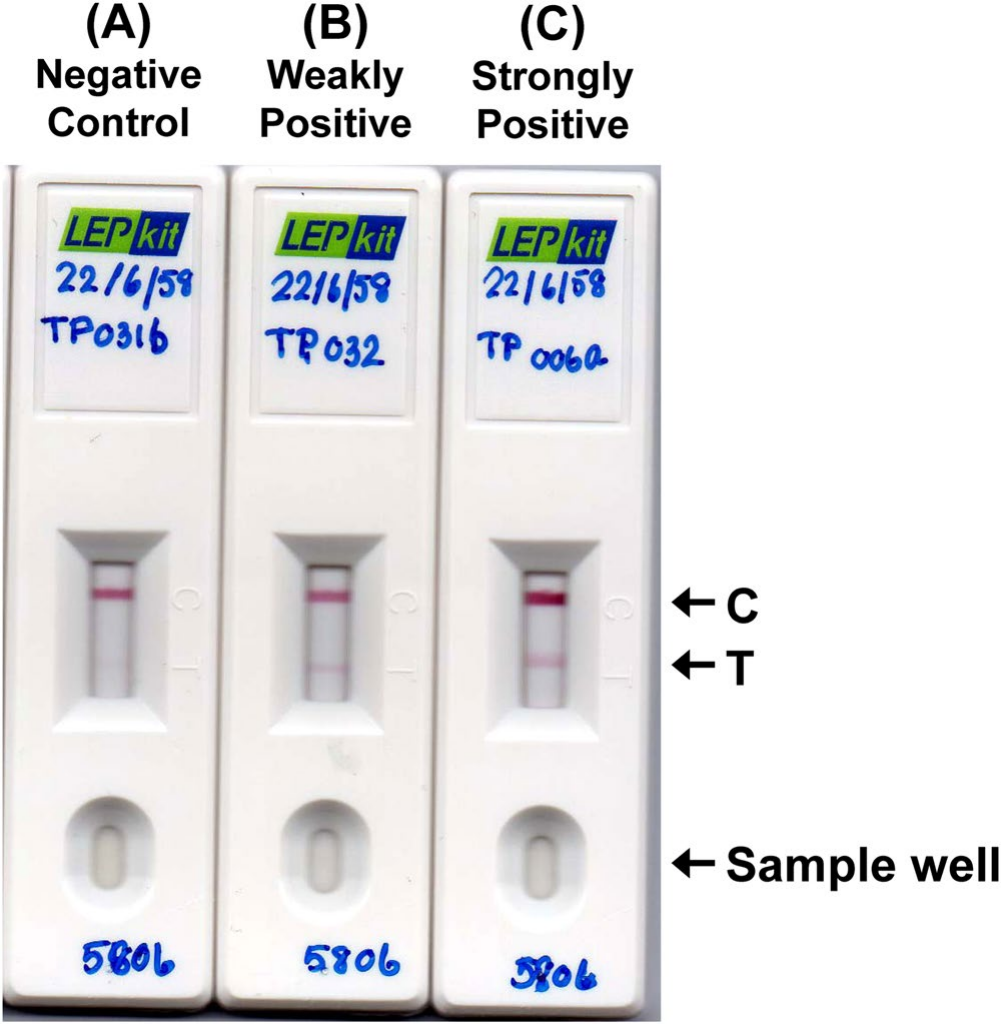
Quality assessment of the LEPkit on detection of acute leptospirosis. (**A**) Negative LEPkit test using serum from a non-leptospirosis patient. (**B**) Weakly positive LEPkit test using serum from a leptospirosis patient with the admission-phase MAT titer <1:400 showed a faint T-band. (**C**) Strongly positive LEPkit test using serum from a leptospirosis patient with the admission-phase MAT titer ≥1:400 showed a strong T-band. C = Controlled line, T = Tested line.

The LEPkit was assembled as detailed in “Materials & Methods”. Theoretically, the serum with anti-leptospiral LPS IgM (i.e. from acute leptospirosis patient) would show two colorized bands at both T- and C- lines, whereas the negative controlled serum would show a positive band at C-line only. We thus evaluated the validity of our in-house LEPkit by using sera from non-leptospirosis and acute leptospirosis with low and high MAT titers. The data showed that there was only one colorized band at the C-line when the non-leptospirosis serum was applied (Fig. 2A). The acute leptospirosis serum with low MAT titer introduced another colorized but faint band at the T-line (Fig. 2B). Moreover, the serum with high MAT titer caused a much greater intense band at the T-line in addition to the controlled C-line (Fig. 2C).

### The stability and reproducibility of LEPkit

**Figure 3:**
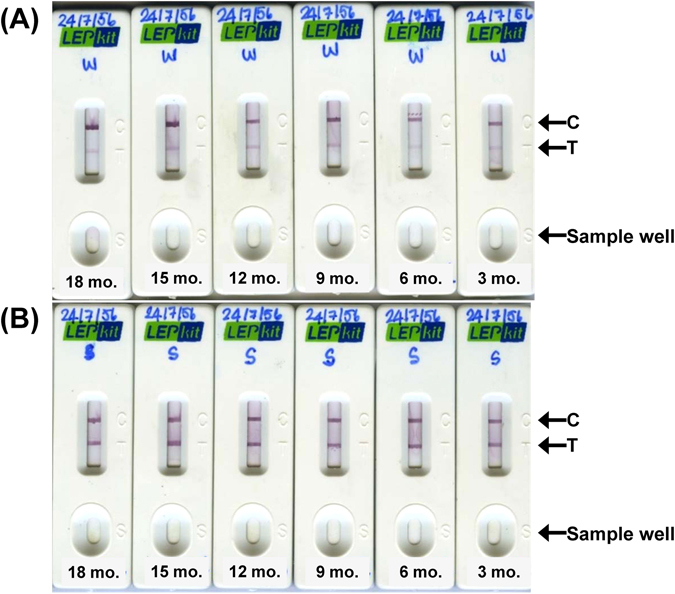
The stability and reproducibility of LEPkit. (**A**) Weakly (W) positive LEPkit test using serum from a leptospirosis patient with the admission-phase MAT titer <1:400 showed a faint T-band. (**B**) Strongly (S) positive LEPkit test using serum from a leptospirosis patient with the admission-phase MAT titer ≥ 1:400 showed an intense T-band. The test was performed using individual LEPkits that were stored at room temperature for 18, 15, 12, 9, 6, and 3 months. C = Controlled line, T = Tested line.

The stability and shelf-life of the storage of LEPkit was evaluated after storage at room temperature for 3, 6, 9, 12, 15, and 18 months, with a set of patients’ samples containing low (<1:400) and high (≥1:400) MAT titers. Note that each test on each sample using each LEPkit was randomly assigned to perform and read by three independent laboratory assistants to evaluate the reproducibility. The data showed satisfactory stability and reproducibility of both weakly positive and strongly positive LEPkit tests (Fig. 3). These data indicated that the LEPkit is stable for at least 18 months after storage at room temperature and the results are reproducible among different laboratory personnel.

### Sensitivity, specificity, positive predictive value (PPV), negative predictive value (NPV), and accuracy of LEPkit

Diagnostic performance of the LEPkit was measured against gold standard culture and single MAT using 77 known leptospirosis and 91 non-leptospirosis samples. The data showed very high sensitivity (97.4%), specificity (94.5%), PPV (93.8%), NPV (97.7%) and accuracy (95.8%) (Table 1).

**Table 1:** Sensitivity, specificity, positive predictive value (PPV), negative predictive value (NPV) and accuracy of IgM/LPS-based immunochromatographic assay (LEPkit) in diagnosis of acute leptospirosis in 77 known cases of acute leptospirosis^a^ and 91 non-leptospirosis cases as compared to gold standard test. (Total number = 168).

		Presence of acute leptospirosis (confirmed by gold standard test^a^)		
		Positive	Negative	
**LEPkit test outcome**	**Positive**	75	5	**PPV = 93.8%** (95% CI = 85.4–97.7%)
	**Negative**	2	86	**NPV = 97.7%** (95% CI = 91.3–99.6%)
		**Sensitivity = 97.4%** (95% CI = 90.1–99.5%)	**Specificity = 94.5%** (95% CI = 87.1–98.0%)	**Accuracy = 95.8%** (95% CI = 91.7–98.0%)

^a^Criteria for diagnosis of acute leptospirosis by gold standard test included: 1) positive leptospiral isolation by culture; or 2) MAT titer ≥1:400 at admission-phase; or 3) positive seroconversion (≥1:100) or 4-fold rising in MAT titer of the paired samples. 95% CI = 95% confidence interval determined by McNemar’s test.

### Comparisons of LEPkit with leptospiral culture and single MAT in detection of acute leptospirosis in new suspected cases

**Figure 4:**
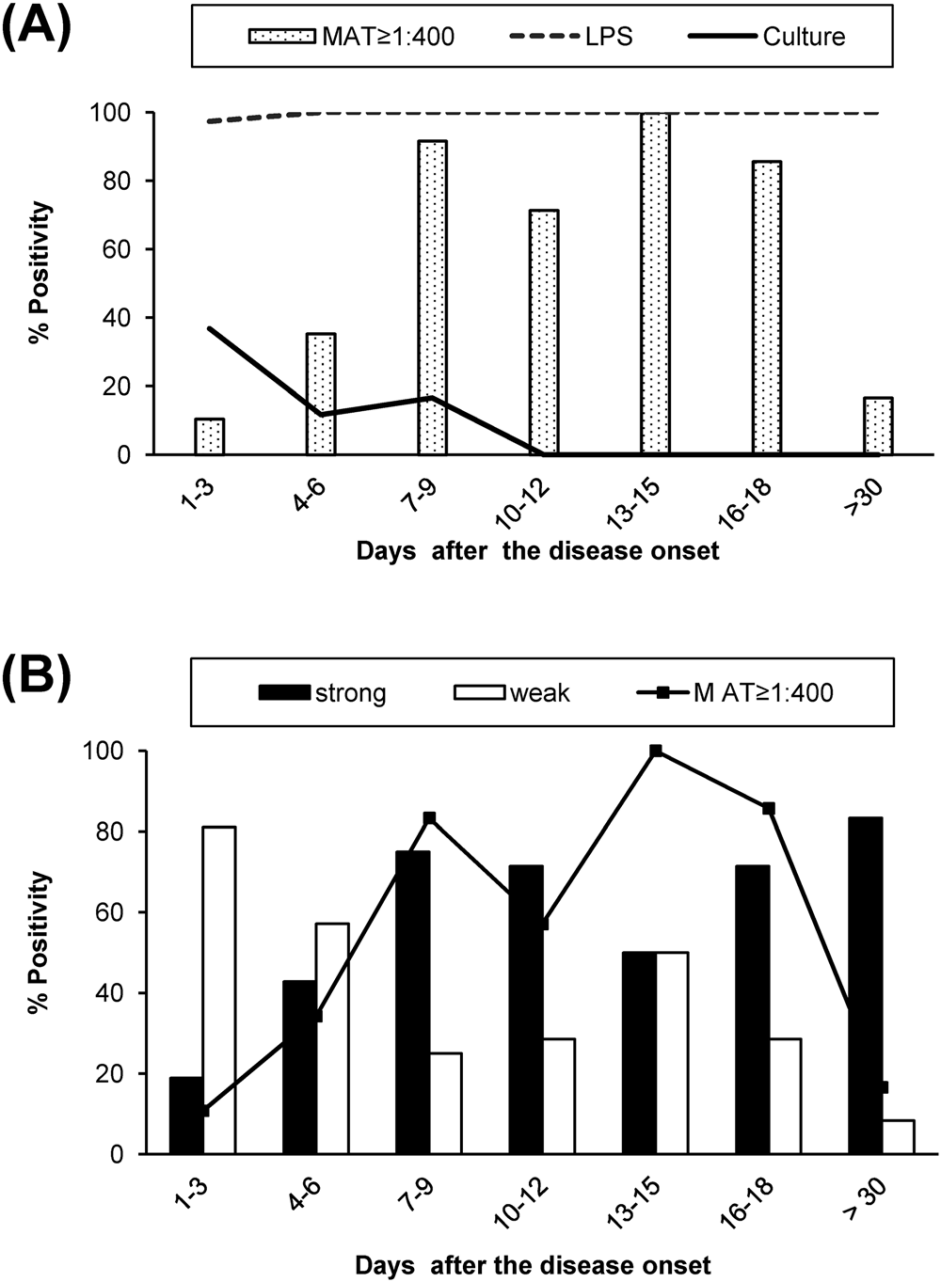
Comparison of the sensitivity of each test for the diagnosis of acute leptospirosis using a single serum sample collected at any time-points during hospitalization. (**A**) Comparisons among the positive tests by culture, single MAT titer ≥1:400, and LPS detection by LEPkit. (**B**) Stratification of strongly and weakly positive LEPkit test as compared to the single MAT titer ≥1:400. (Total number = 135).

From 74 new cases of acute leptospirosis, their serum samples were collected at admission-phase within Day 1–7 after the disease onset (n = 74), whereas subsequent paired samples (n = 61) were collected for seroconversion test. Regardless of the time-points collected (total n = 135), LEPkit was much more sensitive than culture and single MAT (98.5% vs. 15.6% and 35.6%, respectively) (Table 2). When duration of the disease onset was stratified, MAT was most sensitive during Day 7–9, Day 13–15 and Day 16–18 after the disease onset with a sensitivity of 91.7%, 100% and 85.7%, respectively. However, LEPkit offered a high sensitivity (97.4%) since Day 1–3 after the disease onset and maintained a 100% sensitivity throughout the study (up to >30 days) (Table 2). Concerning the degree of positivity of the LEPkit, the weakly positive T-line was observed mostly during early phase of the disease (i.e. Day 1–6 after the disease onset), whereas the strongly positive T-line was mostly observed at later phase (i.e. from Day 7–9 throughout the study – up to >30 days) (Fig. 4).

**Table 2:** Sensitivity of LEPkit in diagnosis of new cases of acute leptospirosis^a^ using a single serum sample collected at any time-points during hospitalization as compared to culture and single MAT. (Total number of samples = 135).

Day after the disease onset	Number of samples tested	% Sensitivity (n) stratified by assay		
		Positive Culture	MAT titer ≥1:400	LEPkit Positive T-line
Day 1–3	38	36.8% (14)	10.5% (4)	97.4% (37)
Day 4–6	34	11.8% (4)	35.3% (12)	100% (34)
Day 7–9	12	16.7% (2)	91.7% (11)	100% (12)
Day 10–12	7	NA	71.4% (5)	100% (7)
Day 13–15	2	NA	100% (2)	100% (2)
Day 16–18	7	NA	85.7% (6)	100% (7)
>30 days	12	NA	16.7% (2)	100% (12)
Unknown^b^	23	4.3% (1)	26.1% (6)	100% (23)
**Total**	**135**	**15.6% (21)**	**35.6% (48)**	**98.5% (133)**

^a^Criteria for diagnosis of acute leptospirosis at any time-points during hospitalization by gold standard test included: 1) positive leptospiral isolation by culture; or 2) MAT titer ≥1:400 at admission-phase; or 3) positive seroconversion (≥1:100) or 4-fold rising in MAT titer of the paired samples. ^b^The disease onset was unclear or was not recorded. NA = Not available.

When only the samples collected at admission-phase were used (n = 74), LEPkit remained much more sensitive than culture and single MAT (98.7% vs. 28.4% and 13.5%, respectively) (Table 3). While the culture and single MAT provided comparable maximal sensitivity of 66.7% at Day 7, LEPkit offered 97.3–100% sensitivity since Day 1–3 through Day 7 (Table 3).

**Table 3:** Sensitivity of LEPkit in diagnosis of new cases of acute leptospirosis^a^ using a single serum sample collected at admission-phase as compared to culture and single MAT. (Total number of samples = 74).

Test	Day after the disease onset	No. positive samples/No. of samples tested	% Sensitivity	95% CI
**Positive Culture**	Day 1–3	14/37	37.8%	22.9–55.2%
	Day 4–6	4/18	22.2%	7.4–48.1%
	Day 7	2/3	66.7%	12.5–98.2%
	Unknown^b^	1/16	6.3%	3.3–32.3%
	**Total (Culture)**	**21/74**	**28.4%**	**18.8–40.2%**
**MAT titer ≥1:400**	Day 1–3	4/37	10.8%	3.5–26.4%
	Day 4–6	2/18	11.1%	2.0–36.1%
	Day 7	2/3	66.7%	12.5–98.2%
	Unknown^b^	2/16	12.5%	2.2–39.6%
	**Total (MAT)**	**10/74**	**13.5%**	**7.0–24.0%**
**LEPkit Positive T-line**	Day 1–3	36/37	97.3%	84.2–99.9%
	Day 4–6	18/18	100%	78.1–100%
	Day 7	3/3	100%	31.0–100%
	Unknown^b^	16/16	100%	75.9–100%
	**Total (LEPkit)**	**73/74**	**98.7%**	**91.7–100%**

^a^Criteria for diagnosis of acute leptospirosis at admission-phase by gold standard test included: 1) positive leptospiral isolation by culture; or 2) MAT titer ≥1:400 at admission-phase. ^b^The disease onset was unclear or was not recorded. 95% CI = 95% confidence interval determined by McNemar’s test.

## Discussion

During an acute phase of leptospirosis when the diagnosis and treatment are extremely critical, the flaws of conventional culture for pathogen isolation and MAT for detection of agglutination antibodies are realized. To date, a limited number of studies have focused on the leptospiral LPS and made use of it for the improved diagnosis or better understanding of the disease pathogenesis and host immunity^37–39^. We described herein the diagnostic performance of a newly developed IgM/LPS-based immunochromatographic assay/device, namely LEPkit, for detection of anti-leptospiral LPS IgM antibody in sera of patients with acute leptospirosis. The capability of our LEPkit imprint with leptospiral LPS antigen to detect circulating IgM in patients’ sera allowed early detection of the leptospiral infection at admission-phase.

Using referenced serum samples of known leptospirosis and non-leptospirosis, LEPkit showed its high sensitivity, specificity, PPV, NPV and accuracy (Table 1). For new cases, when conventional methods were used, only approximately 50% of the samples collected at any time-points and 42% of those collected at admission-phase could be diagnosed by leptospiral culture or a single MAT (Tables 2 and 3). This indicated that a half (or even higher proportion) of the patients would have gone under-diagnosis by gold standard test at admission. Additionally, it is usually delayed to make a proper diagnosis, e.g. after acquiring positive results on prolonged cultivation or positive MAT on the second or paired serum samples. Note that the second peak of high sensitivity of MAT during Day 13–18 (Table 2) for the detection of acute leptospirosis in the serum samples regardless of the time-points collected was most likely due to seroconversion. In contrast to the conventional methods, our data indicated that LEPkit was much higher sensitive and could be used for earlier detection of acute leptospirosis, i.e. during the first few days after the disease onset, whereas the conventional MAT could be used for effective diagnosis later (i.e. Day 7–9 after the disease onset).

Typically, MAT assay is not precise and sensitive enough for early diagnosis of acute infection as a high proportion of these cases have to wait until seroconversion or positive bacterial culture. As a result, most of the patients in endemic regions of leptospirosis are often misdiagnosed as other acute febrile illnesses. The consequence of missed diagnosis or misdiagnosis of acute leptospirosis at an early phase is the possible delay and improper intervention. The diagnosis of acute leptospirosis is even more complicated when the patients are from endemic areas of other infectious diseases where there are increasing number of reports for co-infection/co-incidence of leptospirosis with other endemic infectious diseases, e.g. dengue virus infection^8, 15–17, 19, 20^, malaria^40–44^, and melioidosis^13^.

The use of LEPkit has been proven to be 7.3-time more sensitive than a single MAT titer of ≥1:400 and 3.5-time more sensitive than leptospiral culture for the diagnosis at admission-phase samples (Table 3). Not much is known about immune response to this infectious pathogen. However, the potent cytokine response to the pathogenic strains in human whole blood has been reported^45^. With the delay in accurate diagnosis and appropriate treatment, the patients are at risk of further progression to more severe manifestations and ultimately fatal outcome. Our IgM/LPS-based immunochromatographic assay has shown its great promise for earlier detection of almost all of the suspected cases even with negative culture and MAT. Obvious advantage as demonstrated in this study is that our IgM/LPS immunochromatographic assay can help to alleviate the gap in clinical diagnosis of an early phase of acute leptospirosis, especially for the patients who were actually infected with leptospires but with very low MAT titers during the first few days after the disease onset.

Additional advantages of the LEPkit are that it was not affected by duration of the storage and the personnel who actually handled clinical samples (Fig. 3). And the results of LEPkit assay could be read within only 15-min after introducing the samples to the sample well (S-well) of the kit. The high convenience, stability and reproducibility of the LEPkit should allow its bedside application feasible.

## Materials and Methods

### Ethical approval

This study involving humans was performed in concordance with the recommendation of the Declaration of Helsinki and was approved by the Ethical Review Committee for Research in Humans (Faculty of Medicine, Ramathibodi Hospital, Mahidol University) (ethical approval no. MURA 2552/1798, MURA 2553/298, and MURA 2556/294). The written informed consents were obtained from all the participants.

### Patients and clinical specimens

Initial evaluation of the test sensitivity and specificity was performed using referenced samples, including 77 known cases of acute leptospirosis diagnosed by gold standard test and 91 negative controls. Criteria for the diagnosis of acute leptospirosis by gold standard test were as follows: 1) positive leptospiral isolation by culture; or 2) MAT titer ≥1:400 at admission-phase; or 3) positive seroconversion (≥1:100) or 4-fold rising in MAT titer of the paired samples. The negative controls were healthy blood donors (n = 39) and patients with non-leptospirosis diseases, including syphilis (n = 12), hepatitis (n = 10), dengue virus infection (n = 10), Scrub typhus (n = 10) and melioidosis (n = 10).

In addition, the second set of subjects for further comparisons of the test sensitivity included 74 new cases of acute leptospirosis with age of 33 ± 14.1 years (male:female = 2.6:1) who were presented with acute febrile illness and clinically suspected acute leptospirosis, which was subsequently confirmed by the gold standard test. Their admission-phase samples (n = 74) were collected within Days 1–7 after the disease onset, whereas subsequent paired samples (n = 61) were collected for seroconversion test.

Note that the standard MAT was independently performed at the National Reference Center for Leptospirosis (National Institute of Health, Thailand) with 26 leptospiral serogroups^46^, whereas the leptospiral LPS rapid detection assay (LEPkit) was done in our laboratory. All the sera were stored at −20 °C until used.

### LPS antigen preparation

Briefly, six combined *Leptospira* serovars of local prevalence (including Autumnalis, Bratislava, Canicola, Pomona, Sejroe and Shermani) were grown in liquid Difco^TM^ Leptospira Medium Base Ellinghausen-McCullough-Johnson-Harris (EMJH) medium (Becton Dickinson; Sparks, MD) at 29 °C and then harvested at mid-logarithmic phase (approximately 1 × 10^8^ bacteria/ml). Bacterial cells were lyzed with 0.1 mg/ml proteinase K in 1% sodium dodecyl sulphate (SDS), 1 mM dithiothreitol (DTT) and 0.1 M Tris buffer (pH 8.8) and heat inactivated before dialysis against distilled water. The supernatants of the extracts were collected by centrifugation and stored at −20 °C until used. Protein concentrations were measured by the Bradford’s method, whereas LPS contents were determined by the commercial test kit (Glycoprotein Carbohydrate estimation kit, Pierce; Rockford, IL).

### Initial assessment of anti-leptospiral LPS IgM antibody in patients’ sera for detection of leptospiral LPS by IgM immunoblotting

*Leptospires* were grown in EMJH medium (Becton Dickinson) at 29 °C and then harvested at mid-logarithmic phase (approximately 1 × 10^8^ bacteria/ml), whereas the LPS antigen was prepared as aforementioned. The whole cell and LPS antigens were solubilized in Laemmli’s buffer, heated at 100 °C for 5 min, and resolved in 12.5% sodium dodecyl sulfate (SDS) polyacrylamide gels using a Hoefer Mighty Small II mini-gel apparatus (Amersham Biosciences; San Francisco, CA) with a voltage of 200 V for approximately 1 h. The resolved proteins and LPS were transferred onto a 0.45-µm-thick polyvinylidene fluoride (PVDF) membrane using a Semi-Dry system (TE70; Amersham Biosciences). The immunoblots were initially probed with leptospirosis or non-leptospirosis patient’s serum sample with a dilution of 1:100–1:500 in TBS-T buffer (containing 0.05 M Tris buffered saline (pH 7.4) with 0.05% Tween-20) mixed with 2% skim milk and incubated at room temperature for 1 h. After washing with TBS-T, rabbit anti-human IgM (Dako; Glostrup Denmark) conjugated with horseradish peroxidase was used as the secondary antibody (diluted 1:1,000 in TBS-T with 2% skim milk) and incubated with the membrane at room temperature for 1 h. Immunoreactive bands were then visualized using 3,3′-diaminobenzidine (DAB).

### Design and development of the immunochromatographic assay to detect serum anti-leptospiral lipopolysaccharide IgM (LEPkit)

Leptospiral LPS extracted as aforementioned was used to generate the antigen tested line (T-line), whereas protein A served as the controlled line (C-line). Both lines were immobilized on a nitrocellulose (NC) membrane (AE99, Whatman Schleicher & Schuell; Dassel, Germany) using XYZ 3060 (BioDot; Irvine, CA). The membrane was dried and kept in a dehumidifier cabinet. Immunogold nanoparticles (40-nm, Diagnostic Consulting Network; Carlsbad, CA) conjugated with goat anti-human IgM antibody (Jackson ImmunoResearch; West Grove, PA) (adjusted to 14 µg/ml in 2 mM borate buffer (pH 9.0) containing 1% BSA) were then impregnated (GF33, Whatman Schleicher & Schuell). The conjugated pad (GF33 membrane), NC membrane, sample pad and wicking pad (Whatman) were cut into a 5-mm-wide strip by a Guillotine Cutter (Arista Biologicals Inc.; Allentown, PA) and finally assembled on a plastic back plate. This in-house IgM/LPS-based rapid detection kit (LEPkit) was then sealed and kept at room temperature until used.

### Quality assessment of the LEPkit on detection of acute leptospirosis with low and high MAT titers

The quality of LEPkit was initially assessed using sera from negative controls (non-leptospirosis) and known cases of acute leptospirosis with low (<1:400) and high (≥1:400) MAT titers. Serum (10 µl) from each patient was first spotted into the sample well (S-well) followed by 100 µl phosphate buffered saline (PBS) with 0.05% (v/v) Tween-20 (pH 7.6). Theoretically, anti-LPS IgM in the serum sample of acute leptospirosis patient migrating from the sample well (S-well) through the device would form the complex with immunogold nanoparticles conjugated with goat anti-human IgM antibody. Such complex would then bind to the leptospiral LPS at the tested line (T-line), which would be colorized and visualized as the first band. The excess IgM would further migrate and bind to the controlled line (C-line) immobilized with protein A. The serum with anti-leptospiral LPS IgM (i.e. from acute leptospirosis patient) would show two colorized bands at both T- and C- lines, whereas the negative controlled serum would show a positive band at C-line only. The test would be considered invalid when the C-line showed no band and should be repeated.

### The stability and reproducibility of LEPkit

The stability and shelf-life of the storage of this readily developed device at room temperature was evaluated periodically at 3-month interval (at 3, 6, 9, 12, 15, and 18 months) with a set of patients’ samples containing low (<1:400) and high (≥1:400) MAT titers. Moreover, each test on each sample using each LEPkit was randomly assigned to perform and read by three independent laboratory assistants to evaluate the reproducibility.

### Comparisons of LEPkit with leptospiral culture and single MAT

LEPkit assay was then performed in two independent sample sets comparing to the gold standard leptospiral culture and single MAT. Details of subjects and serum samples of these two sample sets are described above in “Patients and clinical samples”. Leptospiral culture was performed in liquid Difco^TM^ Leptospira Medium Base EMJH medium (Becton Dickinson) at 29 °C for 1–4 months. Positive leptospiral culture was considered when leptospires were observed under a dark-field microscope (Olympus DP70 BX51; Shinjuku, Tokyo, Japan). MAT was performed as detailed in our previous study^46^. Briefly, 50 µl of each serum sample was incubated at room temperature with an equal volume of suspension of live leptospires (approximately 1 × 10^8^ cells/ml) in separate wells of microtiter plates. After 2-h incubation, the maximum dilution titer of serum was considered positive when >50% agglutination was observed under the dark-field microscope (Olympus DP70 BX51).

### Statistical analysis

Diagnostic accuracy of LEPkit was evaluated in comparison with the gold standard test, including leptospiral culture and single MAT. The data are reported as percentage of the positive tests per total number of the samples analyzed as well as 95% confidence interval (CI). The McNemar test was performed to evaluate categorical variables. Sensitivity, specificity, positive predictive value (PPV), negative predictive value (NPV), and accuracy were also calculated.
